# Persistent pollutants and the developing brain: the role of PFAS in neurodevelopmental disorders

**DOI:** 10.3389/fncel.2025.1696173

**Published:** 2025-11-21

**Authors:** Laura Lagostena, Valeria Magnelli, Davide Rotondo, Francesco Dondero

**Affiliations:** 1 Institute of Biophysics (IBF), Italian National Research Council, Genova, Italy; 2 Department of Science and Technological Innovation (DISIT), Università del Piemonte Orientale “Amedeo Avogadro”, Alessandria, Italy

**Keywords:** neuroinflammation, cognitive development, PFOS (perfluorooctane sulfonate), placental transfer, epigenetic modulation, endocrine disruption activity, PFOA (perfluorooctanoate)

## Abstract

Per- and polyfluoroalkyl substances (PFAS) are a diverse class of highly persistent organofluorine compounds, and extensively used in industrial and consumer application. Their environmental ubiquity and bioaccumulation in humans have raised concerns about potential health impacts, particularly on neurodevelopment. This mini-review synthesizes epidemiological and experimental research published between 2020 and 2025 examining prenatal PFAS exposure and neurodevelopmental outcomes in children. Prospective birth cohort studies from Europe, North America, and Asia report subtle but statistically significant associations between higher maternal PFAS levels and a range of neurodevelopmental disorders (NDDs), including autism spectrum disorder (ASD), attention-deficit/hyperactivity disorder (ADHD), cognitive delays (e.g., reduced IQ, language impairments), and behavioral dysregulation. Mechanistic investigations reveal that PFAS can cross the placenta, alter maternal–fetal thyroid and sex-steroid hormone homeostasis, activate inflammatory pathways (e.g., AIM2 inflammasome), disrupt neurotransmitter systems (notably dopaminergic and GABAergic signaling), modulate fetal metabolomic profiles, and induce durable epigenetic modifications. Key methodological challenges include heterogeneity of PFAS mixtures, reliance on single-time-point exposure assessments, variable confounder control (e.g., socioeconomic status, maternal IQ, nutrition, breastfeeding), limited follow-up into later childhood or adolescence, and sparse data on emerging short-chain PFAS analogs. To strengthen causal inference and inform public health interventions, future research should employ longitudinal designs with repeated biomonitoring, standardized neuropsychological assessments, advanced mixture-modeling approaches, comprehensive confounder adjustment, inclusion of vulnerable populations, and focused evaluation of replacement PFAS. Coordinated efforts bridging epidemiology, mechanistic science, and regulatory policy are essential to mitigate PFAS exposure and safeguard neurodevelopmental health in future generations.

## 1 Introduction

Per- and polyfluoroalkyl substances (PFAS) comprise a large class of man-made organofluorine chemicals, numbering in the thousands, that have been in widespread use since the mid-20th century. PFAS owe their utility to a backbone of carbon–fluorine bonds that imparts exceptional thermal and chemical stability, making them effective surfactants and repellents. Historically, legacy long-chain PFAS such as perfluorooctane sulfonate (PFOS) and perfluorooctanoate (PFOA) were extensively deployed in industrial, commercial, and military applications – from non-stick cookware and stain-resistant textiles to grease-proof food packaging, metal plating, aqueous film-forming foams used in firefighting, and fluoropolymer manufacturing (Buck et al., 2011). Because of their prolific use and near-indestructibility, PFAS have become globally ubiquitous environmental contaminants.

They are often termed “forever chemicals” due to their extreme persistence and propensity to bioaccumulate. PFAS pollution now extends from municipal landfills to remote ecosystems: PFOS and other major PFAS are detected in water, soil, wildlife, and are present at low levels in the blood of virtually all people worldwide. Human biomonitoring confirms near-universal exposure – for example, the detection frequency in serum exceeded 98 % for PFHxS, n-PFOS, Sm-PFOS, n-PFOA and PFNA in the 2013–2014 NHANES survey (Calafat et al., 2019), indicating that virtually the entire population had measurable levels of these compounds and underscoring the potential for chronic bioaccumulation in populations.

Growing awareness of PFAS’ environmental persistence and toxicity has prompted recent regulatory and toxicological actions. International authorities now advocate managing PFAS as a chemical class of concern. Certain “long-chain” PFAS have been singled out for strict regulation: notably, PFOS and PFOA (along with related homologues) are now listed as persistent organic pollutants under the Stockholm Convention, effectively banning or restricting their production due to their persistence, bioaccumulative nature, and health hazards. In parallel, PFAS have come under scrutiny by health agencies. The International Agency for Research on Cancer (IARC) recently classified PFOA as possibly carcinogenic to humans (Group 2B, with updated evaluation as Group 1) and PFOS as possibly carcinogenic (Group 2B) (IARC, 2025). Some PFAS are also recognized endocrine disruptors and immunotoxicants, raising concerns about subtler developmental effects (Grandjean et al., 2012). These developments reflect a consensus that PFAS pollution poses a serious public health and environmental risk, warranting precautionary regulation and further study. Against this backdrop, a growing body of epidemiological research suggests that prenatal exposure to PFAS may contribute to neurodevelopmental disorders (NDDs) in children (Bharal et al., 2024). Emerging evidence from prospective birth cohorts in North America, Europe, and Asia indicates that maternal PFAS burdens are associated with modest but measurable increases in the risk of NDD-related outcomes in offspring. Specifically, prenatal PFAS exposure has been linked to higher odds of autism spectrum disorder (ASD) and attention-deficit/hyperactivity disorder (ADHD) diagnoses, as well as subtle reductions in cognitive function – for instance, lower childhood IQ and executive functioning scores in relation to elevated in utero PFAS levels. Other studies report associations with language delays, altered behavior, and general neurodevelopmental impairments, although results across cohorts are not always consistent (see Table 1 and related references). Overall, while effect sizes tend to be small, the convergence of findings strengthens the plausibility that prenatal PFAS exposures can disrupt developmental processes in the fetal brain, contributing to mild NDD phenotypes in early life. This mini-review will synthesize recent epidemiological and experimental evidence on PFAS-related neurodevelopmental toxicity, focusing on outcomes in early childhood (ASD, ADHD, cognitive and behavioral development) and discussing underlying biological mechanisms and public health implications.

**TABLE 1:** Summary of key studies on prenatal PFAS exposure and neurodevelopmental outcomes.

References	Cohort / country	PFAS studied	Neurodevelopmental outcome	Key findings
Itoh et al., 2025	JECS / Japan	PFOS, PFOA, GenX, PFHxS	Developmental delay at age 4	Positive association with deficits in communication and social skills
Eick et al., 2024; Vuong et al., 2021	Atlanta African American Maternal/USA HOME/USA	PFOS, PFOA, PFHxS, PFNA, PFDA, PFUnDA, Me-PFOSA-AcOH	Behavioral problems (ages 3–8)	Higher prenatal PFAS levels associated with increased internalizing and externalizing behavior scores across cohorts
Choi et al., 2024	GUSTO / Singapore	PFOS, PFNA, PFDA	Emotional and executive regulation	Impaired self-regulation linked to prenatal PFAS exposure
Ames et al., 2023	USA	PFOS, PFHxS	ASD diagnosis	Elevated ASD risk with higher maternal PFAS levels
Oh et al., 2021; Oh et al., 2024	South Korea	Multiple PFAS	Autistic traits, gene expression	Placental molecular alterations associated with ASD symptoms
Huang et al., 2024	China	PFOS, PFUnDA	ASD traits, sex steroids, neuro genes	PFAS linked to hormonal and cognitive impairments
Mascari et al., 2025	Italy (Sacco Valley)	PFUnDA, PFHxS	ASRS (Autism Spectrum Rating Scale)	Positive effect independent of IQ and socioeconomic status
Skogheim et al., 2021	MoBa / Norway	PFOS, PFOA	ADHD symptoms	Positive association with ADHD-related symptoms in early childhood
Kim et al., 2023	South Korea	Multiple PFAS	Inattention, hyperactivity	PFAS exposure linked to ADHD traits; sex-specific effects observed
Di Nisio et al., 2022	*In vitro*	PFOA	Dopaminergic toxicity	Damage to developing human dopaminergic neurons
Oh et al., 2024	Placenta and cord blood	Multiple PFAS	Transcriptomics, neurodevelopmental pathways	Neuro gene dysregulation linked to exposure and behavioral traits
Beck et al., 2023	Odense Child Cohort / Denmark	PFOS, PFOA, PFBS	IQ at age 7	Higher maternal PFAS linked to modestly reduced IQ scores
Wang et al., 2023	Shanghai Birth Cohort / China	PFBS, PFOS	Infant IQ, cognitive domains	PFBS associated with lower IQ; prenatal PFOS linked to domain-specific deficits
Forns et al., 2020	Multiple European cohorts	Multiple PFAS	ADHD symptoms	Meta-analysis did not find consistent association with ADHD risk

## 2 Recent epidemiological evidence of PFAS neurodevelopmental impacts

Numerous recent studies have investigated the association between prenatal exposure to PFAS and infant neurobehavioral development, with sometimes conflicting results. Overall, the most recent evidence confirms subtle but significant adverse effects: for example, in a Danish cohort (Odense Child Cohort), high concentrations of PFAS in maternal blood during pregnancy were associated with a slightly lower IQ in children at age 7 (Beck et al., 2023). Although the average decline in IQ observed for individual children is modest, the authors emphasize the possible relevance at the population level given the ubiquitous exposure to PFAS. Similarly, a study conducted in the Shanghai Birth Cohort (∼2000 mother-child pairs) showed inverse associations for specific PFAS: perfluorobutane sulfonate (PFBS, a short-chain “new generation” compound) was associated with a reduction in infant IQ, and prenatal exposure to PFOS was correlated with lower scores in specific cognitive abilities (Wang et al., 2023). However, in this same Chinese cohort, no significant association was found between overall exposure to PFAS mixtures during pregnancy and total IQ at 4 years of age, reflecting heterogeneity in the results. In fact, large epidemiological studies present conflicting results: some large-scale investigations report no correlation or even unexpected “protective” associations (i.e., better scores in more exposed children), probably due to confounding biases related to socio-dietary factors associated with exposure. For example, in the Odense Child Cohort, children with higher blood levels of PFAS at 18 months initially appeared to have slightly higher IQs at 7 years, but this positive effect disappeared after statistically controlling for the benefits of breastfeeding (which increases both infant exposure to PFAS and cognitive development) (Beck et al., 2023). In summary, updated epidemiological evidence suggests a plausible link between prenatal exposure to PFAS and mild neurocognitive deficits (delays in language development, reduced cognitive abilities, attention/hyperactivity problems), but with differences between studies due to relatively low exposure levels, limited samples, and possible confounders. A 2020 European meta-analysis (Forns et al., 2020), for example, found no consistent association with ADHD risk, highlighting inconsistent results across cohorts. On the other hand, some recent studies indicate possible specific effects for individual compounds: a Norwegian study (Skogheim et al., 2021) found an increased risk of ADHD and autism spectrum disorder diagnoses in children exposed to moderate levels of PFOA in utero, while for other PFAS (PFOS, PFDA, PFUnDA) observed inverse associations (lower risk in those most exposed), probably due to uncontrolled confounding. The authors caution that such inverse correlations should not be interpreted as real protective effects, but rather as an indication of residual confounding factors and the need for further research. Overall, the new epidemiological evidence reinforces concerns that prenatal exposure to PFAS, including emerging compounds, may contribute to neurodevelopmental disorders, albeit with mild effects that are difficult to separate from other factors. This literature therefore remains inconclusive and requires larger, more integrated prospective studies to confirm or refute the current findings. Recent large-scale epidemiological reviews have provided an integrated synthesis of PFAS exposure and neurodevelopmental outcomes. In particular, in a recent review summarized 61 studies published between 2008 and 2024, highlighting consistent evidence of subtle but potentially population-relevant effects on cognition, language, and behavior following early-life PFAS exposure. Their analysis also identified major research gaps, including the need to evaluate PFAS mixtures, new-generation compounds, and long-term neuropsychological outcomes. These findings reinforce the epidemiological relevance of PFAS exposure on neurodevelopment and support the need for mechanistic insight (Ames et al., 2025).

### 2.1 General cognitive and behavioral outcomes

Several large-scale cohort studies have documented associations between prenatal PFAS exposure and delays or impairments in broad neurodevelopmental domains such as communication, executive function, emotional regulation, and motor coordination.

One of the most comprehensive investigations to date comes from the Japan Environment and Children’s Study (JECS), a nationwide birth cohort encompassing over 100,000 mother–child pairs. In a recent analysis, Itoh et al. (2025) found that higher maternal blood levels of several PFAS, including PFOS, PFOA, and GenX, were associated with an increased risk of developmental delays in 4-year-old children. The most affected domains included communication skills and social interaction, highlighting the sensitivity of early social-emotional development to chemical exposures.

Several epidemiological studies have linked prenatal PFAS exposure to behavioral and neurodevelopmental outcomes in early childhood. Evidence from multiple U.S. birth cohorts supports the link between prenatal PFAS exposure and early behavioral outcomes. In the HOME Study (Vuong et al., 2021), higher maternal PFHxS concentrations were associated with increased internalizing and externalizing behavior scores in children aged 5–8 years. Similarly, findings from the Atlanta African American Maternal–Child Cohort (Eick et al., 2024) showed that prenatal exposure to PFHxS, PFOS, and PFDA was associated with greater behavioral problems at age 3 years. Collectively, these findings suggest that early-life PFAS exposure may disrupt neurobehavioral development during critical windows of brain maturation. These results suggest that early-life PFAS exposure may influence emotional and behavioral regulation during key stages of childhood development.

Additional support comes from the Singapore GUSTO cohort (Growing Up in Singapore Toward Healthy Outcomes), which evaluated PFAS exposure in relation to early neurobehavioral outcomes. Choi et al. (2024) found that higher prenatal PFAS levels were associated with poorer emotional regulation and reduced executive function skills in toddlers and preschoolers, both of which are critical predictors of later academic performance and social competence.

Collectively, these findings suggest that prenatal PFAS exposure may compromise core cognitive and behavioral functions, with lasting implications for mental health and developmental trajectories. Collectively, these findings suggest that prenatal PFAS exposure may compromise core cognitive and behavioral functions, with lasting implications for mental health and developmental trajectories. In line with the recent comprehensive review by Ames et al. (2025), which integrates epidemiological findings across multiple cohorts and highlights subtle but consistent associations between early-life PFAS exposure and alterations in social, cognitive, and emotional development, our work emphasizes emerging mechanistic insights that may explain these behavioral and cognitive effects.

### 2.2 Autism spectrum disorder (ASD)

Autism spectrum disorder (ASD) is a complex neurodevelopmental condition characterized by deficits in social communication and the presence of restricted, repetitive behaviors. Increasing attention has focused on the potential contribution of environmental factors -particularly endocrine-disrupting chemicals like PFAS- to ASD risk.

Several large epidemiological studies have reported associations between prenatal PFAS exposure and ASD diagnosis or ASD-like traits. In a U.S.-based study, Ames et al. (2023) observed that elevated maternal serum levels of PFOS and PFHxS were associated with significantly higher odds of an ASD diagnosis in children. These findings align with mechanistic studies suggesting that PFAS can disrupt neurodevelopmental pathways involved in social behavior.

In Korea, Oh et al. (2021, 2024) conducted follow-up analyses in two independent cohorts. The 2021 study linked prenatal PFAS exposure to ASD risk, suggesting potential placental or cord-blood molecular alterations, while the 2024 study described maternal–child PFAS exposure relationships. These molecular changes were found to correlate with ASD symptom severity at age 5, suggesting a potential epigenetic mechanism linking prenatal exposure to later behavioral outcomes.

In China, research from the Shanghai Birth Cohort (Wang et al., 2023) provided additional evidence for a connection between prenatal PFAS levels and ASD-related behaviors. Interestingly, the study identified disruptions in sex hormone pathways as potential mediators, raising questions about sex-specific vulnerability to PFAS toxicity in neurodevelopment.

Complementing these findings, Mascari et al. (2025) reported from an Italian cohort that higher maternal PFUnDA concentrations were associated with elevated scores on the Autism Spectrum Rating Scale (ASRS) in children aged 4–7. This association remained significant after controlling for confounders such as maternal IQ and socioeconomic status, strengthening the argument for a direct link between PFAS exposure and ASD risk.

Together, these studies highlight a concerning pattern: PFAS exposure during critical periods of brain development may contribute to the emergence of ASD symptoms, possibly through hormonal, genetic, or inflammatory pathways. Prospective data indicate links with ASD diagnoses and traits (Ames et al., 2023; Mascari et al., 2025), mechanistic evidence from cord blood metabolomics and placental pathways (PPAR, immunità) (Li Z. et al., 2024; Nian et al., 2022; Szilagyi et al., 2020; Wang et al., 2021)and hormonal mediation (Huang et al., 2024).

### 2.3 Attention-deficit/hyperactivity disorder (ADHD)

Attention-deficit/hyperactivity disorder (ADHD) is one of the most common neurodevelopmental disorders of childhood, characterized by persistent patterns of inattention, hyperactivity, and impulsivity. Recent epidemiological evidence suggests that prenatal PFAS exposure may increase the risk of ADHD symptoms through its effects on brain development, particularly in regions involved in attention regulation and executive function.

Data from the Norwegian Mother, Father and Child Cohort Study (MoBa) have been instrumental in elucidating these associations. In two studies (Beck et al., 2023; Skogheim et al., 2020), higher maternal concentrations of PFOS and PFOA were associated with increased ADHD symptoms in school-aged children. Notably, the associations were observed even after adjusting for key confounders, supporting a potential causal relationship.

In Korea, Kim et al. (2023) examined PFAS exposure in relation to behavioral outcomes in a longitudinal birth cohort. They found significant correlations between maternal PFAS levels and increased inattention and hyperactivity symptoms in children, with some indications of sex-specific effects, suggesting that male and female fetuses may differ in their vulnerability to PFAS.

The JECS cohort also contributed relevant findings in this area. In an earlier analysis by Itoh et al. (2022), higher prenatal concentrations of various PFAS compounds were linked to ADHD-like behaviors, reinforcing the emerging consensus that PFAS exposure may interfere with neurobiological circuits involved in attention and self-regulation.

Taken together, these studies support the hypothesis that PFAS may impair attentional control and executive functioning, possibly by disrupting neurotransmitter systems, promoting neuroinflammation, and/or altering neurodevelopmental timing during gestation. Associations with inattention/hyperactivity and behavioral dysregulation have been observed in Norway and Korea (Kim et al., 2023; Skogheim et al., 2021), as well as with externalizing behaviors (e.g., aggression) and sleep problems in U.S. cohorts (Choi et al., 2024).

## 3 Mechanistic insights from human and experimental studies

**FIGURE 1:**
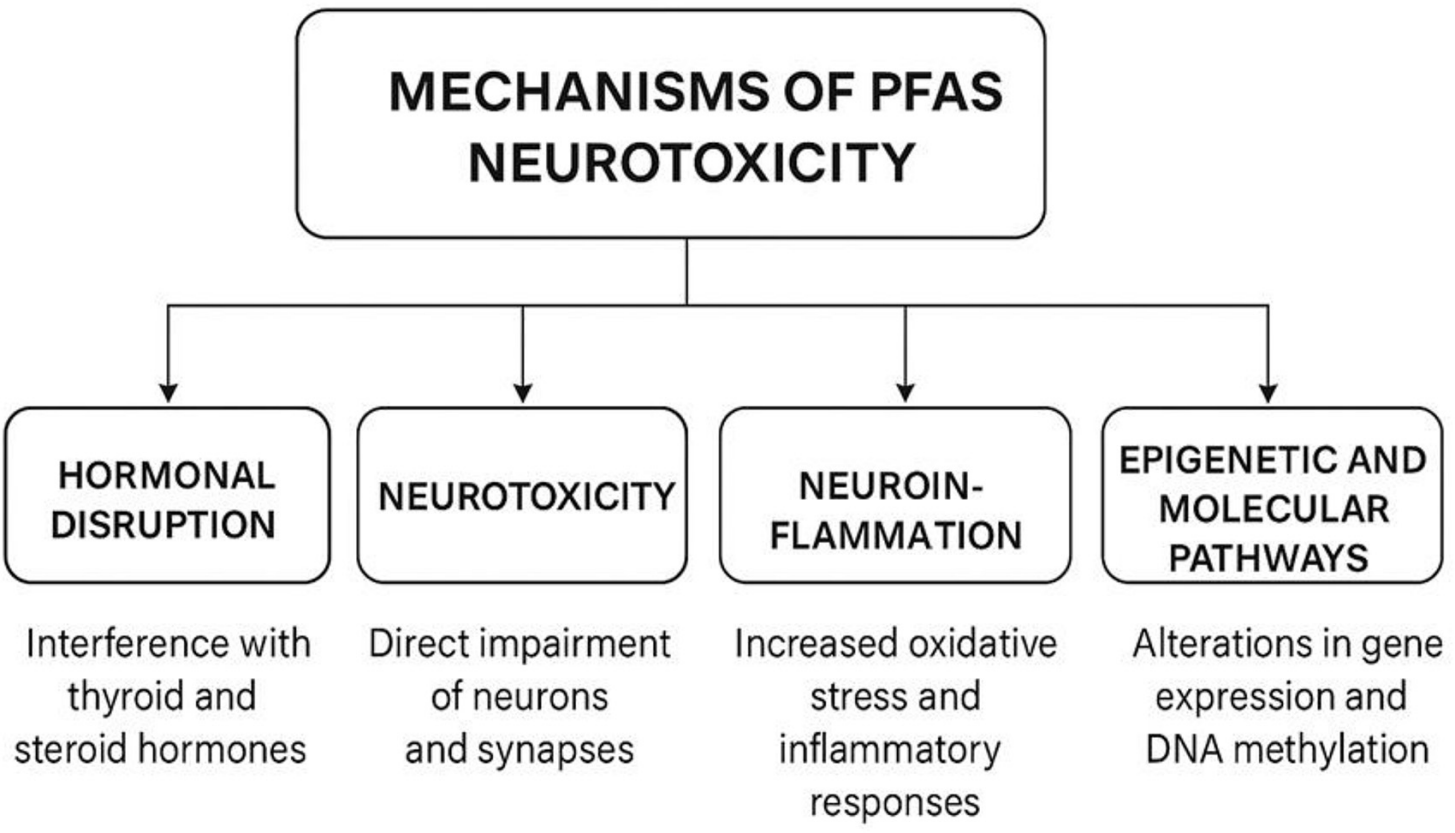
Mechanisms of PFAS and neurodevelopmental disorders.

Understanding the mechanisms by which PFAS impact neurodevelopment is critical for interpreting epidemiological findings and identifying vulnerable populations. While the precise pathways remain under investigation, converging evidence from both human studies and experimental models points to multiple biological mechanisms, including hormonal disruption, neurotoxicity, inflammation, and epigenetic modulation, that may act independently or synergistically to impair early brain development (Figure 1).

The pathways through which PFAS may interfere with neurodevelopment are the subject of intense study, including immunological, neuroendocrine, metabolic, and epigenetic mechanisms. To orient readers among these diverse experimental observations, Table 2 provides a non-exhaustive summary of the main molecular pathways documented to date using *in vitro* and non-human *in vivo* models, specifying the PFAS involved, the experimental model and the key findings. The table highlights recurring patterns across studies rather than attempting to catalogue every mechanism.

**TABLE 2:** Non-exhaustive summary of mechanistic pathways linking prenatal PFAS exposure to neurodevelopmental outcomes.

PFAS and mechanism	Model and exposure	Evidence and key findings
PFOS – PPARg activation and altered neural differentiation	Rat neural stem cells (25–50 nM) and mouse fetuses exposed in utero	*In vitro*, PFOS reduces proliferation and biases stem cells toward neurons and oligodendrocytes via PPARg; UCP2/3 up-regulated. *In vivo*, prenatal exposure elevates PPARg and UCP3 (Wan Ibrahim et al., 2013).
PFOS – GSK-3b/b-catenin signaling	Mouse C17.2 neural stem cells	PFOS decreases pSer9-GSK-3b, activating the kinase; b-catenin and downstream targets are down-regulated, reducing proliferation (Dong et al., 2016).
PFOS/PFOA/PFHxS/PFNA – Oxidative stress and apoptosis	Primary rat hippocampal neurons and astrocytes	PFOS induces ROS, redox imbalance, apoptosis and autophagy; PFOA triggers mitochondrial damage and alters antioxidant capacity; PFHxS and PFNA have distinct effects on total antioxidant capacity (Zhuchen et al., 2023).
PFHxS – NMDA-PKC-ERK/AMPK pathway	Postnatal mice exposed to PFHxS	PFHxS down-regulates GAP-43 and CaMKII via NMDA receptor-mediated PKC–ERK/AMPK signaling, contributing to neurodevelopmental toxicity (Zhuchen et al., 2023).
PFOS – Epigenetic regulation and BDNF	Human neuroblastoma cells; placentas/newborns from PFAS-exposed pregnancies	PFOS causes promoter hypermethylation of BDNF and up-regulates miR-16/-22/-30a-5p, reducing BDNF mRNA and protein (Galea, 2021); maternal PFOS, PFOA, PFHxS and PFDA exposures correlate with DNA-methylation changes in placenta and newborns (Zhuchen et al., 2023).
PFOS/PFOA – GABAA receptor inhibition	S1 neuroblastoma cells	PFOS and PFOA reversibly reduce GABAA receptor-mediated currents, suggesting interference with inhibitory neurotransmission (Lagostena et al., 2024).
PFOS/PFOA – Ca^2+^ channel blockade and synaptic plasticity	Rodent hippocampal slices; *in vivo* LTP studies	PFOS and PFOA block voltage-gated Ca^2+^ channels, reduce field EPSPs and long-term potentiation; nifedipine mitigates PFOS effects (Li S. et al., 2024).
PFAS mixture (prenatal/perinatal) – Developmental outcomes	Pregnant rats consuming PFAS-contaminated water (758.6 ng/L or 3.8 mg/L)	Offspring show reduced anogenital distance and body weight, delayed reflex acquisition and blunted sex differences in locomotion; no major effects on anxiety, depression or memory (Marchese et al., 2024).
Lactational PFOS exposure – Memory and synaptic plasticity	Mice exposed to 1 mg/kg PFOS via nursing	Early PFOS exposure impairs spatial memory and social recognition in middle-aged mice and is linked to reduced synaptic plasticity proteins (Ninomiya et al., 2024).
PFOS/PFOA/PFHxS – Neuronal network activity	Rat cortical cultures on micro-electrode arrays	Acute or chronic exposure to PFOS, PFOA or PFHxS causes limited changes in spike rate and network synchrony; only high-dose BPA or other bisphenols show pronounced effects, while PFAS have minimal impact (Van Melis et al., 2025).
PFOS (+/− vitamin A) – Social behavior and dopamine transporter	Zebrafish larvae exposed to 1–5 mM PFOS with or without vitamin A	PFOS increases inter-individual distance and reduces physical contacts; it down-regulates the dopamine transporter and induces apoptosis genes. Vitamin A partially reverses these effects (Jiang et al., 2025).
PFHxA – Sex-specific cerebellar changes	Pregnant mice given PFHxA (0.32 or 50 mg/kg) during gestation and lactation	PFHxA produces sex-dependent transcriptional changes, increases Purkinje cell number and alters microglial morphology, while down-regulating oligodendrocyte genes (Plunk et al., 2025).
Paternal PFAS mixture exposure – Epigenetic effects in sperm and offspring	Male mice exposed 18 weeks to a mix of five PFAS; gene expression assessed in offspring	Exposure produces ∼2 861 differentially methylated regions in sperm enriched for neurodevelopment and Wnt signaling; offspring show altered liver and fat gene expression (Maxwell et al., 2024).

### 3.1 Emerging molecular mechanisms

Studies on women of childbearing age have also found that both “historical” PFAS (such as PFOA and PFOS) and emerging PFAS alter the profile of circulating cytokines, potentially creating an inflammatory state: in particular, higher levels of certain PFAS have been associated with an increase in Th1 and Treg cytokines (e.g., TGF-β) and a decrease in anti-inflammatory Th2 cytokines (e.g., IL-10) (Nian et al., 2022). Since a pro-inflammatory intrauterine environment is a known risk factor for neurodevelopmental alterations, the immunotoxic effect of PFAS represents a pathway of interest (e.g., chronic activation of inflammatory mediators such as IL-1β could interfere with fetal brain maturation). On the endocrine side, PFAS are also classified as endocrine disruptors: they can bind to nuclear receptors and disrupt key hormonal axes for neuronal development. During pregnancy, PFAS cross the placenta and have been shown to alter maternal-fetal thyroid function (by reducing the synthesis or bioavailability of thyroid hormones) (Drover et al., 2019). This is critical, as adequate maternal thyroxine levels are essential for neuronal migration and myelination in the fetus; mild gestational hypothyroxinemia induced by PFAS can result in cognitive deficits in children. Similarly, some PFAS affect sex hormone levels during pregnancy: a recent study documented alterations in maternal sex steroid concentrations (estrogens, androgens) in relation to PFAS exposure, with differentiated effects depending on the compound and fetal sex (Rivera-Núñez et al., 2023). Such hormonal imbalances could modulate female-male brain differentiation, offering an explanation for the possible sex-specific effects observed in some epidemiological studies. Another identified molecular target is the placenta: PFAS can accumulate in placental tissue and interact with peroxisome proliferator-activated receptors (PPARs), disrupting normal placental vascularization, nutrient transport, and immune modulation processes (Szilagyi et al., 2020; Wasel et al., 2023). This could indirectly compromise fetal neural development through chronic hypoxia or alterations in the release of trophic factors from the placenta. On a metabolic level, metabolomics approaches are beginning to reveal the biochemical alterations associated with prenatal exposure to PFAS. A large study (Li Z. et al., 2024) has identified metabolomic signatures in umbilical cord blood correlated with maternal PFAS levels, mainly involving amino acid, lipid, and neurotransmitter pathways. In particular, exposed newborns had disrupted concentrations of certain neurotransmitter precursors and vitamin cofactors involved in the synthesis of dopamine and glutamate. This suggests that PFAS may also affect neurodevelopment by modulating neuronal metabolism and brain biochemistry in utero, for example by altering the availability of amino acids essential for neurotransmission or by inducing oxidative stress at the mitochondrial level (as evidenced by alterations in the carnitine shuttle and membrane lipids in exposed newborns) (Li Z. et al., 2024). Finally, an emerging field of great interest is that of PFAS-induced epigenetic modifications. Several studies indicate that exposure during the prenatal and perinatal stages can alter DNA methylation and other epigenetic markers in fetal tissues (e.g., cord blood), with potential long-term repercussions on gene expression (Perng et al., 2022). For example, birth cohorts have observed associations between PFAS levels and lasting changes in the methylation of genes involved in nervous system development and metabolism. Some of these epigenetic effects show sex dependence: one study found that PFOS during pregnancy correlated with abnormal methylation patterns in neurodevelopmental genes only in male infants, suggesting different epigenetic vulnerability between the sexes. These early-induced epigenetic alterations can persist for years and contribute to subsequent neurobehavioral outcomes; it is even hypothesized that certain epigenetic “marks” from PFAS may escape normal embryonic reprogramming processes and be maintained in germ cells, potentially transmitting a predisposition to neurodevelopmental disorders to future generations. Although transgenerational transmission in humans remains to be proven, this evidence highlights how PFAS can leave a long-lasting biological imprint. In summary, the most recent research outlines a multifactorial scenario: PFAS act through multiple molecular pathways, including immune-inflammatory dysfunction, hormonal imbalances (thyroid and steroid), metabolic alterations (lipid and neurotransmitter profile), and epigenetic reprogramming. These integrated immuno-neuroendocrine mechanisms provide a plausible biological basis for epidemiological findings and constitute priority areas for the future. Methodological limitations and research gaps-despite the growing number of studies, there are still some big methodological limitations that can affect how we interpret the current data. One big issue is that most of the studies are observational and have a limited follow-up period. Many studies assess neurodevelopmental outcomes only in childhood (often between the ages of 5 and 8), while some disorders only become apparent later: for example, executive function deficits emerge more reliably after the age of 5, and formal diagnoses of ADHD or autism spectrum disorders are often made during school age. The prevalence of early assessments may contribute to inconsistencies between studies, as latent behavioral phenotypes may escape premature detection. It is therefore necessary to extend the longitudinal assessment of exposed children, with follow-up during school age and adolescence, to capture any late effects of PFAS on neurodevelopment. Related to this is the issue of the diagnostic tools used: to date, many studies have used general developmental questionnaires or parent-reported behavior scales, which have limited sensitivity for specific domains (language, cognitive functions, etc.) and may introduce noise into the data. A recent systematic review of language in children exposed to PFAS (Stübner et al., 2023) found no consistent effects, suggesting that the use of non-specific tests may have masked subtle deficits. Looking ahead, it is essential to adopt more targeted and standardized tools, such as clinical neuropsychological tests conducted by specialists, to accurately assess aspects such as language, sustained attention, working memory, and adaptive behavior. The use of objective measures (clinical observations, individual cognitive tests) instead of questionnaires alone will reduce the risk of misclassification of neurodevelopmental outcomes. Another common limitation is the assessment of PFAS exposure limited to a single period (typically a maternal blood sample during pregnancy). Although this provides an indication of fetal exposure, it does not capture ongoing postnatal exposures: PFAS persist in the home environment and can be ingested through food, contaminated water, and dust, potentially affecting child development after birth. Few studies have so far integrated both prenatal and childhood measurements; in the Odense Child Cohort, for example, PFAS was also measured in the blood of children at 18 months, but the interpretation of the postnatal contribution was complex due to the intertwining with breastfeeding (Beck et al., 2023). In general, cohorts with repeated measurements (pregnancy, early childhood, school age) are needed to distinguish the cumulative effects of early exposure in different windows of vulnerability. Furthermore, the high correlation between different PFAS (often co-occurrence of PFOA, PFOS, PFHxS, etc., in the same individuals) makes it difficult to isolate the effect of a single compound. More sophisticated statistical approaches, such as models for mixed exposures (e.g., quantile g-computation, mixture analysis), are beginning to be applied, but require large samples to have sufficient statistical power. Many past studies with few participants (<200) risk having produced unstable estimates or false associations (in particular, anomalous “protective” results may reflect statistical instability in small samples). Control of confounders also varies: factors such as socioeconomic status, maternal IQ, nutrition, and family environment influence neurodevelopmental outcomes and may be associated with PFAS levels (e.g., fish consumption or prolonged breastfeeding, which increase both PFAS exposure and, respectively, the intake of beneficial nutrients and cognitive stimulation of the child). Recent studies show that factors such as maternal education, equality, a stimulating home environment, and the duration of exclusive breastfeeding can mitigate the effects of neurotoxic contaminants. Therefore, it is essential to incorporate these covariates and analyze any interactions (for example, the effect of PFAS may be greater in children with diets lacking in neuroprotective nutrients or who are not breastfed). Another gap is the poor representation of vulnerable populations: many cohorts consist of families of medium-high socioeconomic status and areas that are not highly polluted, limiting generalizability. Future studies should include more diverse samples (disadvantaged socioeconomic groups, communities with high exposure through drinking water or industry) to clarify whether there are subgroups of children who are more susceptible to the effects of PFAS. Finally, a crucial methodological issue concerns the scope of exposures considered: most research to date has focused on so-called “legacy” substances (PFOA, PFOS, PFHxS, PFNA), while there are a growing number of emerging or substitute PFAS for which data are scarce. Compounds such as GenX (HFPO-DA), ADONA, PFBA, PFBS, and other shorter-chain or modified molecules are now widely used in place of PFOA/PFOS, and toxicological studies indicate that they are not without effects. For example, an experimental investigation in zebrafish compared GenX and PFBS, observing neurobehavioral toxicity in larval development (motor hyperactivity, alterations in dopamine levels) in both, with even more pronounced effects for PFBS (Wasel et al., 2023), suggesting that these alternatives may also interfere with developing neural circuits. Epidemiologically, the limited data available are cause for concern: the aforementioned Shanghai study found that maternal PFBS was among the main PFAS associated with a decline in IQ in children. In addition, short-chain PFAS and some new fluorinated ethers show placental transfer efficiency equal to or greater than traditional compounds: in a Chinese cohort (Wasel et al., 2023), PFBA (perfluorobutanoic acid) and an alternative chlorinated PFAS (6:2 Cl-PFESA) had cord/mother ratios of ∼1 or greater, indicating neonatal blood concentrations equal to or greater than maternal concentrations (Cai et al., 2020). This implies that alternative substances easily cross the placental barrier and can reach the fetus in biologically relevant doses. Including emerging PFAS in biomonitoring campaigns and cohort studies is therefore a priority, as is assessing their long-term toxicity profile. In conclusion, to fill the current gaps, the following is recommended: (a) longer longitudinal studies with multidimensional neuropsychological assessments up to adolescence; (b) the use of specific diagnostic tools and objective measures for the various domains of neurodevelopment; (c) the design of studies capable of integrating prenatal and postnatal exposures and applying mixture models, improving the control of confounders; (d) the extension of analyses to new PFAS and high-risk populations. These methodological improvements will strengthen the ability to identify even moderate causal effects of PFAS on neurodevelopment and guide effective public health strategies in the future.

### 3.2 Hormonal disruption and brain development

Hormonal signaling, particularly involving thyroid and steroid hormones, is essential for orchestrating neurodevelopmental processes such as neuronal proliferation, migration, synaptogenesis, and myelination. During gestation, the developing brain is highly sensitive to fluctuations in maternal hormone levels, making endocrine-disrupting chemicals like PFAS a significant concern.

Several studies have documented the ability of PFAS to disrupt steroidogenesis and thyroid hormone regulation. For example, Huang et al. (2024) demonstrated that prenatal PFAS exposure was associated with altered steroid hormone profiles in cord blood, specifically showing significant reductions in estradiol and testosterone. These hormones play key roles in brain sexual differentiation and the establishment of neural circuits involved in emotional and cognitive regulation. The observed hormonal alterations were correlated with neurobehavioral abnormalities in early childhood, suggesting a plausible mechanistic link.

Additionally, PFAS have been shown to interfere with thyroid hormone homeostasis. Thyroxine (T4) and triiodothyronine (T3) are critical for neurodevelopment, particularly in regulating gene expression during brain maturation (Du et al., 2024). Although results across human studies are somewhat inconsistent, likely due to differences in PFAS species, exposure levels, and population characteristics, there is a general trend indicating that PFAS exposure is associated with lower circulating thyroid hormone levels. Such disruptions may impair neuronal differentiation and migration during fetal development, increasing susceptibility to neurodevelopmental disorders.

An increasing number of studies suggest that neurobehavioral outcomes associated with prenatal exposure to PFAS may differ between males and females, likely due to the interaction between endocrine disruption and sex-dependent trajectories of brain development. In Asian cohorts, higher maternal PFAS levels have been associated with alterations in emotional and executive regulation during early childhood, with some indications of sex-dependent effects, such as stronger associations observed in males in cord-blood metabolomic analyses (Li Z. et al., 2024); however, the evidence from (Choi et al., 2024; Kim et al., 2023) does not clearly document sex-specific interactions. These patterns are consistent with evidence on ASD-related traits, in which the maternal–fetal steroidal milieu appears to partially mediate the association between PFAS exposure and neurodevelopmental outcomes, with potential sex-dependent modulation (Huang et al., 2024).

At the mechanistic level, several PFAS compounds interfere with both thyroid homeostasis and steroidogenesis during pregnancy (Drover et al., 2019; Du et al., 2024; Rivera-Núñez et al., 2023). Alterations in estrogen and androgen levels during critical developmental windows may affect the sexual differentiation of the brain and the formation of limbic–cortical circuits, contributing to sex-divergent neurobehavioral phenotypes (Huang et al., 2024). Moreover, placental involvement mediated by PPAR activation, leading to immunometabolic and nutrient transport reprogramming, represents an additional mechanism of sex-specific susceptibility, given the well-documented placental dimorphism in response to inflammatory stress (Szilagyi et al., 2020).

Finally, multi-omic and molecular evidence indicates that prenatal PFAS exposure is associated with dysregulation of neurodevelopmental and immune-related pathways (Nian et al., 2022; Wang et al., 2021), as well as with metabolomic alterations in cord blood involving amino acid and lipid metabolism, which may reflect a potential impact on fetal metabolic programming (Li Z. et al., 2024). It is plausible that such biochemical and epigenetic alterations interact with the hormonal milieu in a sex-dependent manner, thereby amplifying differences in risk between males and females.

### 3.3 Neurotoxicity and neuroinflammation

An additional axis of toxicity involves immune activation and inflammatory signaling in the developing nervous system. PFAS can act as immunotoxicants at both peripheral and central levels, eliciting neuroinflammation that is detrimental to brain tissue (Ehrlich et al., 2023; Martano et al., 2025). As summarized in a recent mini-review by Martano et al. (2025), multiple *in vivo* and *in vitro* studies have shown that PFAS exposure provokes robust inflammatory responses across various organs, including the central nervous system.

A defined molecular mechanism is the activation of the AIM2 inflammasome by PFOS, which triggers IL-1β secretion and pyroptosis; mechanistically, PFOS drives mitochondrial DNA release via a Ca^2 +^-PKC-NF-κB/JNK-BAX/BAK axis, and Aim2^–/–^ mice show attenuated PFOS-induced inflammation and tissue injury (Wang et al., 2021). AIM2-deficient mice exhibit attenuated inflammation and tissue damage after PFOS exposure (Li S. et al., 2024). Systemic inflammation in peripheral organs can also weaken the blood–brain barrier, amplifying neurotoxicity; with increasing severity and duration of systemic inflammation, BBB permeability to solutes rises and innate immune cell entry is facilitated, compounding central inflammation (Galea, 2021). Thus, prenatal PFAS exposure may set off central and peripheral immune responses that together create a neuroinflammatory milieu detrimental to brain development.

Many of the above mechanisms converge on oxidative stress. PFAS increase reactive oxygen species, inhibit antioxidant enzymes such as superoxide dismutase, and open mitochondrial permeability transition pores (Zhuchen et al., 2023). In fetal brains, PFAS exposure is associated with mitochondrial DNA damage, lipid peroxidation and activation of pro-apoptotic cascades that eliminate developing neurons and oligodendrocytes (Li S. et al., 2024). Oxidative stress can also suppress Nuclear factor erythroid 2-related factor 2 (NRF2), an antioxidant transcription factor, thereby reducing Brain Derived Neurotrophic Factor (BDNF) levels and exacerbating inflammation (Kebieche et al., 2025).

Several PFAS directly affect neuronal function and synaptic communication. A key mechanism is the dysregulation of intracellular calcium: for example, PFOS and PFOA cause a pathological increase in Ca^2+^ in neurons both *in vitro* and *in vivo*, promoting its entry through membrane channels (such as L-type Ca^2+^ channels) and its release from storage organelles (endoplasmic reticulum and mitochondria). Excess neuronal Ca∧2+ alters electrical excitability and abnormally activates Ca^2+^-dependent signaling pathways, with cascading effects: defects in action potential propagation, altered neurotransmitter release and activation of enzymes that damage the cell are observed (Starnes et al., 2022).

Di Nisio et al. (2022) provided compelling evidence that PFOA exposure impairs dopaminergic neurons across multiple stages of *in vitro* development. Their study demonstrated increased neuronal cell death, impaired neurite outgrowth, synaptic deficits, and altered differentiation patterns, all of which are key features of disrupted neurodevelopment. Given that dopaminergic dysfunction is implicated in both ADHD and ASD, these findings lend biological plausibility to the epidemiological associations observed between PFAS and these disorders.

Recently, PFOS and PFOA have been shown to significantly and reversibly reduce GABA_a_ receptor-mediated currents in human neuronal-like cells *in vitro* (Lagostena et al., 2024) and in *Xenopus laevis* oocytes expressing human α1β2γ2 GABA receptors (Tukker et al., 2020), suggesting a possible mechanism by which PFAS may interfere with the GABAergic system. These studies consistently demonstrated that acute exposure to PFOS and PFOA significantly alters GABAergic signaling. In addition, Tukker et al. (2020) examined the impact of these compounds on spontaneous neuronal network activity using multielectrode array recordings, further confirming the disruptive effects of PFAS on key neurophysiological processes. Given the critical role of GABAergic transmission and coordinated neuronal network activity in the development of cognitive and behavioral functions, these findings provide important mechanistic insights into how PFAS exposure may contribute to neurodevelopmental disorders such as ADHD and ASD.

### 3.4 Epigenetic and molecular pathways

Emerging data also suggest that PFAS may influence neurodevelopment through epigenetic mechanisms—altering gene expression without changing the underlying DNA sequence. These effects can have lasting impacts on developmental programming and may partly explain individual differences in susceptibility to PFAS-related outcomes.

In a recent transcriptomic analysis, Oh et al. (2024) reported that prenatal PFAS exposure was associated with altered expression of neurodevelopment-related genes in placental and cord blood samples. The affected genes were involved in critical pathways regulating synaptic formation, neuronal signaling, and immune function. These findings support the hypothesis that PFAS may exert developmental toxicity by disrupting gene regulatory networks during gestation.

Importantly, individual genetic variation appears to modulate vulnerability to PFAS-induced neurodevelopmental effects. In a genome-wide interaction study, Huang et al. (2024) identified specific gene variants related to steroidogenesis and inflammation that modified the relationship between prenatal PFAS exposure and cognitive outcomes in children. These results underscore the complex interplay between environmental exposures and host genetics, suggesting that some individuals may be more susceptible to PFAS toxicity due to their genetic background.

## 4 Limitations of current evidence

While the growing body of epidemiological and experimental studies provides compelling support for an association between prenatal PFAS exposure and adverse neurodevelopmental outcomes, several limitations must be considered when interpreting the findings. These methodological and conceptual challenges highlight the need for cautious interpretation and underscore the importance of refining study designs in future research.

Variability in PFAS mixtures and timing of exposure assessment is one of the most significant obstacles to drawing consistent conclusions across studies. PFAS are a diverse class of chemicals with differing toxicokinetic profiles, biological activity, and environmental persistence. Most studies focus on a limited number of legacy PFAS (e.g., PFOA, PFOS), while fewer assess emerging compounds such as GenX or short-chain alternatives. Moreover, exposure is typically measured at a single time point, often during mid-pregnancy, which may not capture critical windows of susceptibility or reflect cumulative exposure over time. This heterogeneity complicates cross-study comparisons and limits the generalizability of findings.

Residual confounding remains an inherent limitation of observational studies. Although many cohorts adjust for a broad range of covariates, including maternal education, income, smoking, and diet, unmeasured or inadequately measured factors, such as household chemical exposures, psychosocial stress, or parental neuropsychiatric history, may influence both PFAS exposure and neurodevelopmental outcomes. These potential confounders can introduce bias and make it difficult to attribute observed associations solely to PFAS.

Outcome assessment is another source of variability. Studies differ in how neurodevelopment is measured, ranging from standardized clinical assessments to parent-reported questionnaires, behavioral checklists, or educational records. While each approach has value, differences in sensitivity, specificity, and diagnostic rigor can affect the strength and interpretation of observed associations. Additionally, outcomes are often assessed at different developmental stages, from infancy to school age, further complicating comparisons.

Sex-specific effects, though frequently observed, are not always consistently analyzed or interpreted. Several studies report stronger associations in either males or females, reflecting potential sex-based vulnerabilities to endocrine disruption and neurodevelopmental perturbation. However, stratified analyses are not uniformly conducted, and few studies are adequately powered to examine sex differences with statistical robustness. This represents a missed opportunity to understand differential susceptibility and tailor public health recommendations accordingly.

Finally, reverse causation and exposure misclassification are important concerns in observational research. Reverse causation, where a pre-existing condition influences exposure measurement, is less likely in prospective birth cohorts but cannot be entirely excluded. Exposure misclassification, particularly when relying on single-point biomonitoring, may underestimate true exposure variability and attenuate associations. Nonetheless, longitudinal designs with repeated biological sampling and rigorous outcome assessments are helping to mitigate these limitations.

In summary, while the evidence base linking PFAS exposure to neurodevelopmental risk is rapidly expanding, addressing these methodological challenges is essential for improving causal inference. Future studies should aim to incorporate repeated measures of PFAS exposure, standardized neurodevelopmental outcomes, genetic susceptibility data, and more refined confounder adjustment to strengthen the validity and interpretability of findings.

## 5 Future research directions

To advance our understanding of the neurodevelopmental risks posed by PFAS and to strengthen causal inference, future research must adopt more integrative, mechanistically informed, and policy-relevant approaches. Several key priorities have emerged:

Exposome-wide approaches: Given that PFAS rarely occur in isolation, future studies should adopt exposome-wide frameworks that consider the cumulative effects of multiple environmental exposures, including other endocrine-disrupting chemicals, air pollutants, and nutritional factors. This holistic perspective will help disentangle the contribution of PFAS within the broader context of prenatal environmental risk.

Longitudinal cohort studies with extended follow-up: While early childhood outcomes have been the primary focus of most studies to date, the long-term impacts of PFAS on cognitive, emotional, and social development remain understudied. Prospective cohort studies with neurodevelopmental follow-up into adolescence and beyond are essential to capture later-emerging deficits, such as learning disabilities, executive dysfunction, and mental health conditions. These studies should incorporate standardized, age-appropriate assessments across multiple domains.

Mechanistic research using human-relevant models: To bridge the gap between epidemiological associations and biological plausibility, mechanistic studies are needed that employ advanced *in vitro* systems. Human-derived neural stem cells, brain organoids, and microphysiological platforms provide powerful tools to investigate how PFAS influence neuronal differentiation, synaptic architecture, and the formation of functional brain circuits. These advanced *in vitro* systems also enable high-throughput screening of individual PFAS compounds and complex mixtures. Complementarily, traditional animal models remain necessary and essential for assessing the broader effects of prenatal PFAS exposure on cognitive processes such as learning and memory, attention and executive function, emotion regulation and stress response, sensory processing, decision-making and impulse control—domains that require integrated behavioral and neurophysiological evaluation.

Gene–environment interaction studies: Emerging evidence suggests that genetic variation may influence individual susceptibility to PFAS-induced neurotoxicity. Large-scale studies integrating genomic and transcriptomic data with environmental exposure profiles will be critical for identifying vulnerability loci and elucidating the molecular pathways involved. Such insights could pave the way for precision public health strategies targeting high-risk subgroups.

Translational research and policy integration: Given the ubiquity of PFAS exposure and the mounting evidence of their potential harm, research must inform and keep pace with regulatory action. Efforts to translate findings into risk assessment frameworks and exposure guidelines are essential, particularly in light of recent initiatives such as the European Union’s proposed REACH restrictions and the U.S. EPA’s draft limits on PFAS in drinking water. Collaborative engagement between scientists, policymakers, and public health agencies will be key to ensuring that evidence-based regulations effectively protect vulnerable populations, especially pregnant women and children.

Finally it should be pointed out that in assessing the causal link between PFAS exposure and neurodevelopmental disorders, it is crucial to acknowledge the substantial heterogeneity of this chemical class, which comprises thousands of congeners with distinct physicochemical, kinetic, and toxicological properties (Buck et al., 2011; Calafat et al., 2019). Co-exposure to multiple compounds and the widespread use of short-chain or ether-based fluorinated substitutes (e.g., GenX/HFPO-DA) complicate cross-study comparisons and make any extrapolation from a single PFAS to the entire class precarious. In this mini-review, we therefore adopt an intentionally broad approach aimed at highlighting convergent risk patterns and plausible mechanisms, while referring to compound-specific analyses for the definition of dose–response relationships and windows of susceptibility (Cai et al., 2020; Wasel et al., 2023).

Given the remarkable diversity among PFAS, we recommend that future cohort studies adopt a congener-comparative design with repeated biomonitoring windows, explicitly including emerging substitutes such as HFPO-DA, PFBS, PFBA, and 6:2 Cl-PFESA (Cai et al., 2020; Wasel et al., 2023). Such an approach, combined with statistical models for chemical mixtures, will help minimize the risk of overgeneralization and misleading conclusions.

## 6 Conclusion

The growing body of scientific evidence increasingly supports a connection between prenatal exposure to PFAS and a broad spectrum of neurodevelopmental disorders. This relationship is reinforced by consistent findings across diverse populations spanning multiple countries, varied study designs, and a wide range of neurodevelopmental outcomes. Importantly, these epidemiological observations are underpinned by plausible and increasingly well-characterized biological mechanisms, such as hormonal disruption, neuroinflammation, oxidative stress, and epigenetic modifications, that provide critical insights into how PFAS may contribute to altered brain development.

As these “forever chemicals” persist and continue to circulate ubiquitously in the global environment, human exposure remains widespread and inevitable, raising significant concerns about the long-term implications for public health, particularly for vulnerable populations such as fetuses and young children. The early developmental stages represent critical windows of susceptibility, during which disruptions to neurodevelopmental processes can have enduring consequences on cognitive, emotional, and behavioral functioning throughout life.

Given the pervasive nature of PFAS contamination and the mounting evidence linking exposure to adverse neurodevelopmental outcomes, it is imperative to strengthen preventive public health strategies aimed at reducing exposure, especially among pregnant individuals and young children. Such measures should include enhanced regulatory controls, improved environmental monitoring, safer alternatives to PFAS-containing products, and targeted educational campaigns to raise awareness about potential sources of exposure.

Simultaneously, advancing scientific research remains crucial to fully elucidate the complex pathways through which PFAS affect brain development. This involves integrating longitudinal epidemiological studies with mechanistic investigations using human-relevant models, alongside the exploration of gene–environment interactions that may influence individual susceptibility, in parallel with neurophysiological and behavioral studies in animal models. A deeper understanding of these dynamics will enable the development of more effective risk assessment frameworks, inform public health policies, and ultimately contribute to protecting neurodevelopmental health across populations.

In summary, while challenges remain, the current evidence strongly implicates PFAS as important environmental contributors to neurodevelopmental disorders. Addressing this issue through coordinated research, policy, and prevention efforts is essential to safeguard the cognitive and behavioral health of future generations and reduce the burden of neurodevelopmental impairments worldwide.
